# Mind in Crisis, Ovary in Question: A Case of Anti-N-Methyl-D-Aspartate (NMDA) Receptor Encephalitis Associated With Ovarian Teratoma

**DOI:** 10.7759/cureus.84935

**Published:** 2025-05-27

**Authors:** Hasiba Mahmoud, Mohammedelfateh Adam, Azriny Khalid

**Affiliations:** 1 Obstetrics and Gynaecology, University Hospital Waterford, Waterford, IRL; 2 Obstetrics and Gynaecology, University Hospital Galway, Galway, IRL

**Keywords:** anti-nmda receptor encephalitis, autoimmune encephalitis, benign mature cystic teratoma, multidisciplinary approach, psychiatric manifestations

## Abstract

Anti-N-methyl-D-aspartate (NMDA) receptor encephalitis is a rare but serious autoimmune disorder. It is often associated with ovarian teratomas and mimics psychiatric or infectious conditions, leading to frequent misdiagnosis. Early recognition and treatment are crucial for better outcomes. We present the case of a 31-year-old woman who developed sudden behavioural changes, seizures, and cognitive decline. Initial tests showed no clear cause. The cerebrospinal fluid analysis confirmed anti-NMDA receptor antibodies, and imaging revealed an ovarian teratoma. Immunotherapy alone failed, and tumour removal was necessary for recovery. Her condition improved significantly after surgery. Our case highlights the difficulty of diagnosing autoimmune encephalitis in young women with unexplained neuropsychiatric symptoms. Recognising paraneoplastic causes and using a multidisciplinary approach are essential for timely intervention. Immunotherapy can help, but it may not be enough without tumour removal. Autoimmune encephalitis requires prompt diagnosis and aggressive treatment. Failure to identify and treat underlying causes can lead to severe complications or delayed recovery. This case emphasises the importance of considering anti-NMDA receptor encephalitis in young women with new-onset neuropsychiatric symptoms. Early cerebrospinal fluid analysis and imaging can confirm the diagnosis and guide treatment decisions.

## Introduction

Anti-N-methyl-D-aspartate (NMDA) receptor encephalitis is a rare autoimmune disorder that was first described in 2007 by Dr. Josep Dalmau and colleagues [1]. It is a severe illness and is also a cause of acute neuropsychiatric manifestations such as psychosis, amnesia, seizures, autonomic instability, and even sometimes complications with body movements [2].

Teratomas, or dermoid cysts, are the most common ovarian germ cell tumours [3]. Different studies have asserted that nearly 7.8-59% of young females with ovarian teratomas develop this disease since the tumours induce an immune reaction against the NMDA receptor [4]. Autoantibodies against NMDA receptors in the central nervous system cause the disease, and they cause synaptic dysfunction and neuroinflammation [5]. Ectopic neural tissue in ovarian teratomas contains NMDA receptors and initiates an autoimmune attack. Autoantibodies produced by the immune system in these patients cross the blood-brain barrier and target neuronal NMDA receptors that cause significant neurological impairment [6]. Viral infections, herpes encephalitis, also play a role in the disease through epitope spreading and immune activation [7].

According to the consensus criteria proposed by Graus et al., a probable diagnosis of anti-NMDA receptor encephalitis can be established when all three of the following conditions are met: (1) at least four out of six core symptom groups (psychiatric or cognitive dysfunction, speech abnormalities, seizures, movement disorders or abnormal posturing, reduced level of consciousness, and autonomic instability or central hypoventilation) appear within three months; (ii) supportive findings from investigations, such as an abnormal electroencephalography (EEG) or cerebrospinal fluid (CSF) abnormalities (pleocytosis or oligoclonal bands); (iii) other causes are ruled out [8].

Early diagnosis can prevent symptoms from worsening, so physicians can diagnose the disease by CSF examination for NMDA receptor antibodies, brain imaging (CT and MRI), and EEG [9,10]. NMDA is more often treated with immunotherapy with corticosteroids or intravenous immunoglobulin (IVIG) and plasmapheresis. Ovarian teratoma patients need tumour resection [11]. Delayed diagnosis may enhance the risk of serious complications, with studies reporting that approximately 9.5% of patients experience persistent severe deficits or death, underscoring the importance of early detection and treatment [4,10].

Epidemiology and aetiology of anti-NMDA receptor encephalitis

Anti-NMDA receptor encephalitis rate is estimated at 1.5 cases per million people every year [12]. It comes under the most prevalent antibody-mediated encephalitis, which is primarily seen to affect young women, and a significant number of cases stem from ovarian teratomas, which act as a key trigger for the immune response [13]. In a Chinese study with a large cohort, 220 confirmed cases were analysed, which identified psychosis (82.7%) and seizures (80.9%) as the most common presenting features [14]. Relapse rate differs across different studies at 17.3% [14], 12% [10], and 12-25% in others [4]. The incidence of associated tumours and ovarian teratomas was 38% [10], lower in some studies (20-40%) with heterogeneity of screening protocols [12].

The aetiology of anti-NMDA receptor encephalitis is most commonly due to immunoglobulin G (IgG) autoantibodies directed against the GluN1 (NR1) subunit of the NMDA receptor, resulting in receptor internalisation and synaptic dysfunction [4]. Molecular mimicry is the most common mechanism because NMDA receptors are present in ectopic neural tissue of ovarian teratoma, which triggers an autoimmune response [15]. Herpes simplex virus (HSV) infection is also considered a trigger in some patients [13]. The course of the disease is variable across populations, with lower rates of intensive care unit (ICU) admission in Asian populations (51.1%) than in Western reports (>70%), possibly reflecting earlier diagnosis and intervention [12].

In this context, we present the case of a 31-year-old previously healthy woman who developed unexpected neuropsychiatric symptoms, including behavioural changes, seizures, and cognitive decline. Despite an inconclusive workup and treatment for suspected infectious encephalitis, further testing revealed anti-NMDA receptor encephalitis associated with an ovarian teratoma. This case highlights the diagnostic challenges of autoimmune encephalitis and the importance of considering paraneoplastic causes in young women presenting with acute psychiatric and neurological symptoms.

## Case presentation

A 31-year-old Sudanese female patient, residing in Ireland, presented to the Emergency Department with acute behavioural and neurological disturbances following emotional distress triggered by media coverage of the recent earthquake in Syria. She was a trained psychologist and a mother of two, with no prior personal or family history of medical, psychiatric, or autoimmune illnesses. The onset of symptoms occurred 10 days before admission, characterised by insomnia, nightmares, increased religious preoccupation, and paranoid ideation. While no formal psychometric assessment was conducted at presentation, symptom severity was clinically significant and prompted hospital referral.

On day one of hospitalisation, she experienced a generalised tonic-clonic seizure lasting approximately five minutes, accompanied by tongue biting and a prolonged postictal state. On examination, her vital signs were all within the normal range. A comprehensive mental state examination and neurological examination were performed. The patient was fully aware of time, place, and person, cooperative, and displayed normal speech and thought processes without hallucinations or delusions. She had no focal deficits, normal cranial nerve function, and intact motor and sensory responses. Routine laboratory investigations, including haematology, metabolic panels, inflammatory markers, and infectious disease screening, were unremarkable (Table 1). Despite the absence of an identifiable infection, we empirically started her on intravenous acyclovir at 10 mg/kg every eight hours and intravenous ceftriaxone 2 g once daily due to concern for infectious encephalitis. She continued both medications for a total of seven days until infectious screening results returned negative.

**Table 1:** Blood investigations RPR: rapid plasma reagin; TPHA: treponema pallidum hemagglutination assay

Test	Patient Values	Units	Reference Ranges
C-reactive protein	3.9	mg/L	0.0 – 5.0
WBC	7.3	x10⁹/L	4.0 – 10.0
RBC	4.2	x10¹²/L	3.8 – 4.80
Hemoglobin	12.9	g/dL	12.0 – 15.0
Hematocrit	0.38	L/L	0.36 – 0.46
Mean corpuscular volume	91.3	fL	83.0 – 101.0
Mean corpuscular hemoglobin	29.7	pg	27.0 – 32.0
Mean corpuscular haemoglobin concentration	34.3	g/dl	31.5– 36.0
Platelets	237	x10⁹/L	150 – 400
Neutrophils	4.89	x10⁹/L	2.0 – 7.00
Lymphocytes	2.83	x10⁹/L	1.0 – 3.00
Monocytes	0.72	x10⁹/L	0.20 – 1.00
Eosinophils	0.47	x10⁹/L	0.02 – 0.50
Basophils	0.08	x10⁹/L	0.02 – 0.10
Urea	4.6	mmol/L	2.50 – 7.80
Sodium	139	mmol/L	135.0 – 145.0
Potassium	4.7	mmol/L	3.50 – 5.30
Chloride	104	mmol/L	95.0 – 108.0
Creatinine	61	µmol/L	45.0 – 84.0
Estimated glomerular filtration rate	>90	mL/min/1.73m²	>90
Alanine transaminase	28	U/L	5.0 – 33.0
Total Bilirubin	8.5	umol/L	2.0 – 21.0
Alkaline Phosphatase	77	IU/L	30.0 – 130.0
Gamma-glutamyl transferase	39	U/L	6.0 – 42.0
Total Protein	71	g/L	60.0 – 80.0
Albumin	48	g/L	35.0 – 50.0
Clacium	2.54	mmol/L	2.20 – 2.60
Phosphorous	1.1	mmol/L	0.8 – 1.5
Magnesium	0.8	mmol/L	0.7 – 1.0
HIV Ag/Ab	Not detected		
Hepatitis B Surface Antigen	Not detected	_	_
Hepatitis C Antibody	Not detected	_	_
Syphilis Serology (RPR/TPHA)	Not detected	_	_

Deterioration and diagnostic workup

On day three, the patient`s condition had worsened significantly, with the emergence of confusion, repetitive speech, and increased agitation. By day five, she developed auditory and visual hallucinations, necessitating transfer from the neurology unit to the psychiatric department for further evaluation. Upon her arrival, a comprehensive mental state examination (MSE) was attempted, but it proved difficult due to her agitated and chaotic presentation. Though a complete assessment of thought content was impossible, she was reported to have paranoid thoughts. Auditory and visual hallucinations were observed, and cognitive function appeared impaired. No suicidal ideation was reported. She required intramuscular haloperidol 5 mg and lorazepam 2 mg with minimal change to her level of agitation.

**Figure 1:**
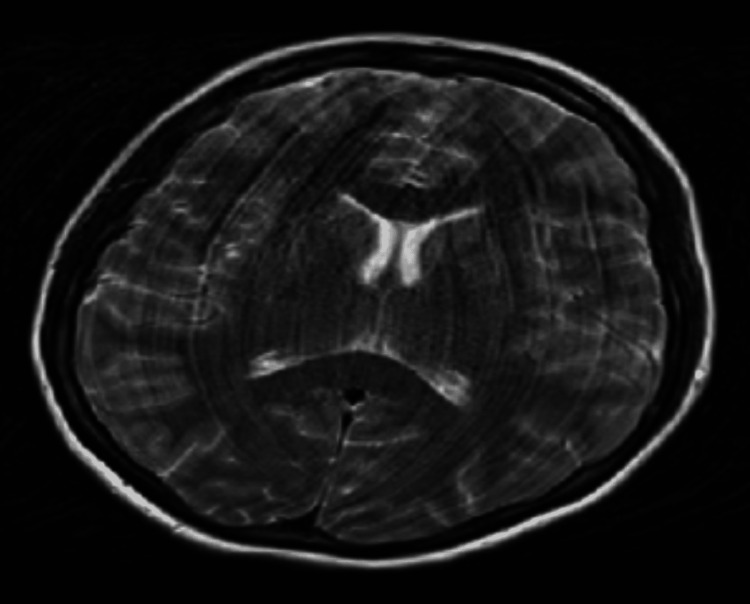
MRI of the brain shows no abnormality

An MRI of the brain was requested, which revealed no abnormalities (Figure 1). A lumbar puncture was performed the following day, yielding normal opening pressure, cell counts, and glucose and protein levels. These normal findings are commonly observed in the early stages of anti-NMDA receptor encephalitis, which can complicate the diagnostic picture.

Given her rapidly worsening condition, a broad differential was considered, including primary psychiatric disorders (e.g., psychotic or severe mood disorders), infectious encephalitis (e.g., HSV encephalitis, bacterial or fungal infections), and autoimmune encephalitis (e.g., anti-NMDA receptor encephalitis, Hashimoto’s encephalopathy).

On day 8, an EEG revealed focal encephalopathy, raising concerns for HSV encephalitis or an autoimmune process. However, HSV polymerase chain reaction (PCR) from CSF was negative, prompting further investigation. The patient's CSF analysis confirmed the presence of anti-NMDA receptor IgG antibodies, establishing a definitive diagnosis of anti-NMDA receptor encephalitis.

**Figure 2:**
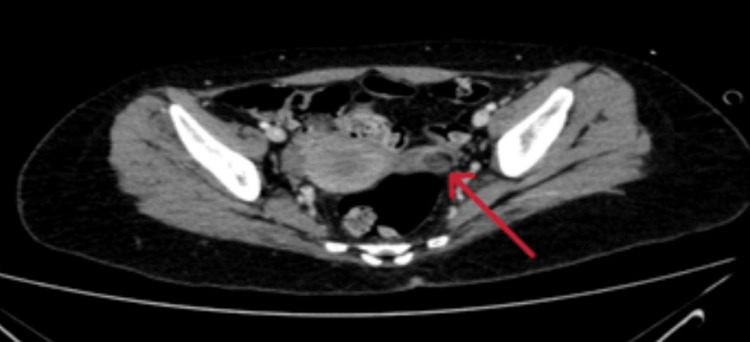
CT scan of the pelvis shows 11 mm solid cystic lesion on the left ovary

Given the strong association between ovarian teratomas and anti-NMDA receptor encephalitis, a CT scan of the abdomen and pelvis was performed on day 10, revealing a focal fat-containing lesion 11 mm in keeping with an underlying teratoma/dermoid (Figure 2). This finding confirmed the paraneoplastic autoimmune origin of the disease. Therefore, she was transferred to the Gynaecology Department for further management.

Therapeutic intervention and clinical course

On day 11, the patient was immediately started on intravenous methylprednisolone 1 g once daily for five days and intravenous immunoglobulin (IVIG) at 0.4 g/kg/day for five consecutive days. However, her clinical response was limited, with ongoing hallucinations, disorientation, and agitation, prompting escalation to second-line immunotherapy. We discontinued IVIG and started her on rituximab 1 g intravenously on day 14, with a second dose planned after 14 days by standard treatment protocols. Despite immunotherapy, her neurological and psychiatric symptoms persisted, reinforcing the urgency of tumour removal as a therapeutic strategy.

**Figure 3:**
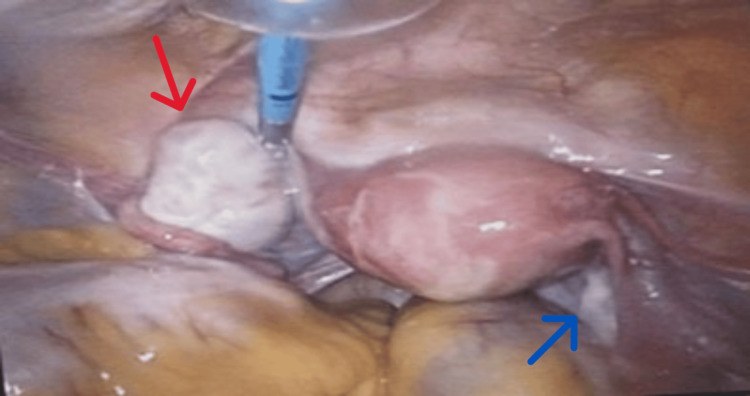
Intraoperative laparoscopic image demonstrating a normal right ovary (blue arrow) and a left ovary with a small cyst (red arrow)

**Figure 4:**
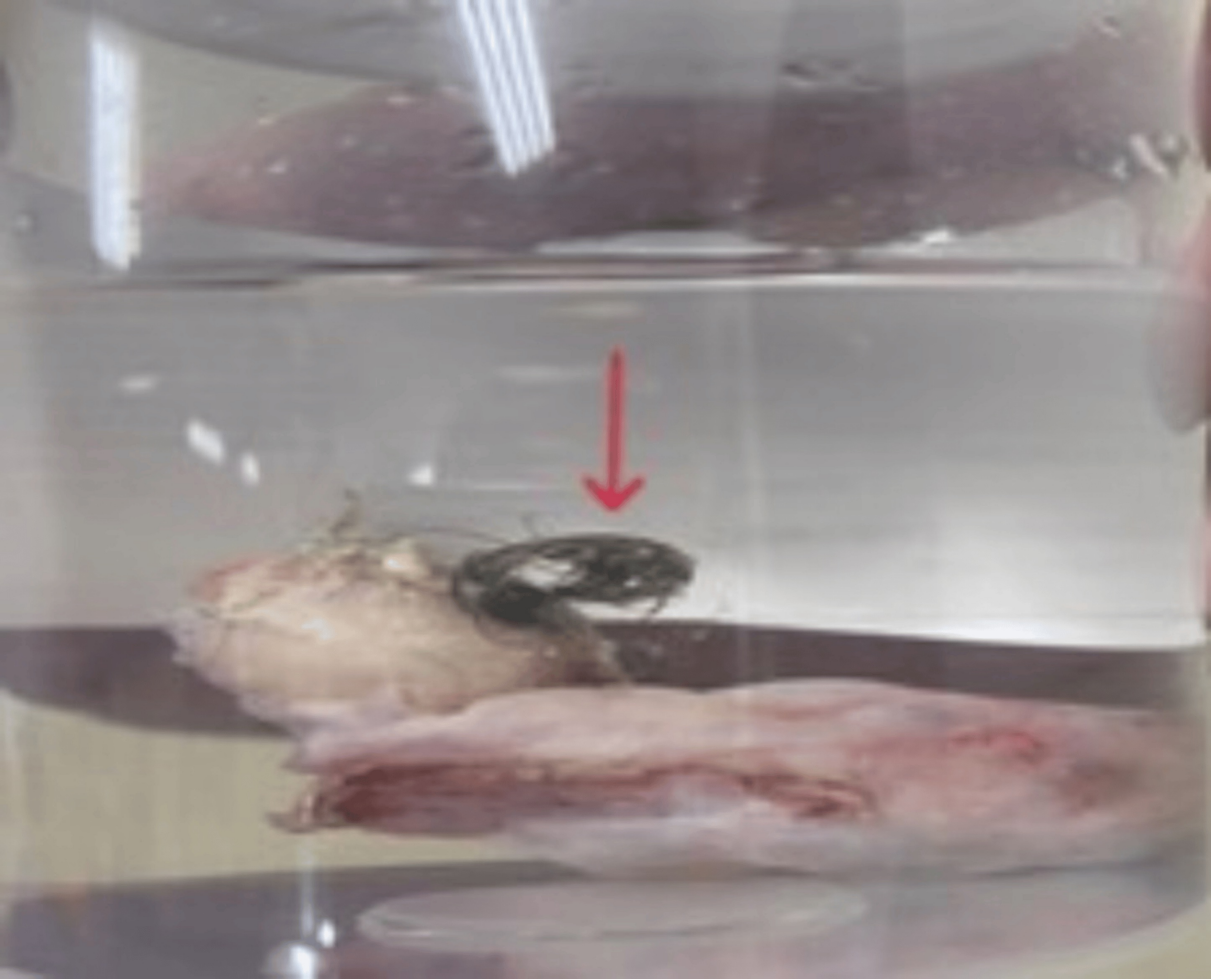
Gross specimen of an ovarian teratoma in a container, demonstrating hair content (red arrow)

On day 32, after multidisciplinary discussions and stabilisation, the patient had a laparoscopic left oophorectomy performed by a consultant gynaecologist. Oophorectomy was chosen over cystectomy due to the lesion's intramedullary location, which makes complete cyst removal difficult and increases the risk of incomplete excision (Figures 3, 4). The histopathological examination revealed an intact ovarian cyst measuring 25 × 10 × 20 mm. The specimen included ovarian tissue with functional cysts as well as a benign dermoid cyst (mature cystic teratoma). The successful removal of the affected ovary eliminated the autoimmune trigger associated with the ovarian teratoma.

Postoperatively, the patient remained in the hospital for continuous monitoring. Initially, she experienced mild fatigue and drowsiness. By day 35 (day 3 postoperative), her agitation and hallucinations had resolved. She regained full orientation and coherent speech after five days postoperatively. Within one week, she achieved functional independence and continued rehabilitation through physical and occupational therapy. Objective neurocognitive evaluation was conducted using the Mini-Mental State Examination (MMSE®) (PAR, Inc., Florida, United States) during recovery. Scores demonstrated progressive improvement in attention, memory, and orientation domains.

On discharge, follow-up with Neurology, Psychiatry, and Rehabilitation services was arranged to support her continued improvement. Her recovery program includes a multidisciplinary approach aiming at optimising neurological and functional outcomes. Physical therapy was initiated to restore motor coordination, while occupational therapy focused on cognitive and functional reintegration. In addition, psychotherapy and nutritional support were provided to enhance overall recovery and promote general well-being.

Outcome and follow-up

The patient demonstrated full neurological and functional recovery within two months of tumour removal. She was discharged home on a tapering course of oral corticosteroids (prednisolone 40 mg daily, tapered over six weeks) and levetiracetam 500 mg twice daily for seizure prophylaxis.

She was followed up at one month, three months, and six months post discharge. At each visit, she remained clinically stable with no recurrence of seizures, psychosis, or cognitive symptoms. At the six-month follow-up (the last recorded visit), she had returned to her baseline level of functioning, resumed her role as a mother and professional, and reported no neuropsychiatric complaints. No further immunosuppressive therapy was deemed necessary. Levetiracetam was continued for seizure prophylaxis at 500 mg twice daily, with a plan to taper under neurology guidance after 12 months if no seizures occurred.

## Discussion

Diagnosing anti-NMDA receptor encephalitis is challenging because it mimics psychiatric and infectious disorders. Misdiagnosis and delayed treatment are common. The patient in the current report showed behavioural changes as she experienced sleep disturbances, paranoia, and religious preoccupation. These symptoms often lead to a psychiatric diagnosis, and a tonic-clonic seizure marked a critical shift; however, doctors did not immediately consider an autoimmune cause. No focal neurological deficits were present, and the initial laboratory results appeared normal. These factors made diagnosis even more difficult [16,17].

Despite progressive neurological findings, such as agitation and confusion, early investigations were inconclusive. MRI findings were normal, which is not unusual in early anti-NMDA receptor encephalitis. Studies have shown that MRI abnormalities are seen in fewer than half of cases and, when present, are often nonspecific [14]. Some studies report that typical MRI changes occur in only about 30% of patients, especially when scans are performed at the time of disease onset. However, these findings can change over time, and their absence early on does not rule out the diagnosis [10,12].

Diagnosis in this patient hung on CSF analysis, with the presence of anti-NMDA receptor antibodies being detected. It is the demonstration of a high index of suspicion and vigorous workup in the face of normal investigations not reflecting the clinical course [18]. Anti-NMDA receptor encephalitis pathophysiology is driven by autoantibodies that disrupt synaptic function and target NMDA receptors in the brain. Disruptions lead to a cascade of neurological and psychiatric symptoms that evolve from behavioural and cognitive disturbances to autonomic dysfunction, seizures, and profound neurological impairment [1].

In this patient, the presence of an ovarian teratoma played a pivotal role. These tumours harbour ectopic neural elements with NMDA receptor expression that trigger a cross-reactive immune response to receptors in the central nervous system. This linkage of gynaecologic pathology with profound neurological compromise highlights the need for a full systemic evaluation in patients with suspected autoimmune encephalitis [5,19].

The necessity of performing a gynaecological examination in female patients presenting with unexplained neuropsychiatric symptoms is of utmost importance. The prevalence of ovarian teratomas and anti-NMDA receptor encephalitis has been described to be high, with nearly half of the female patients presenting with an underlying tumour [20]. The case is characteristic of the immediate impact of tumour removal on the course of the disease. A combination of immunosuppressive therapy and tumour removal is key to successful recovery. Though initial treatment was with IVIG and corticosteroids, the patient did not show notable improvement, and thus, the necessity to initiate second-line treatment with rituximab. The failure of neuropsychiatric symptoms to resolve with immunotherapy was a clear indicator of the necessity for therapeutic intervention [21]. A breakthrough was achieved with the surgical resection of the ovarian teratoma, which led to dramatic improvement in cognitive and functional capacity. This observation is by clinical findings suggesting that removal of the tumour in paraneoplastic diseases is not merely additive but usually central to achieving complete recovery [20].

The therapeutic strategy in anti-NMDA receptor encephalitis is, by nature, multidisciplinary. The failure of the patient to respond to first-line immunotherapy lends credence to available literature that indicates such patients with a pre-existing teratoma need both tumour removal and immunotherapy for the best results. Research by Dalmau et al. has indicated that when both approaches are used, between 75% of patients recover completely, while those who are treated with immunotherapy alone suffer long-lasting illness and higher relapse rates [1]. The current case also illustrates that patients who undergo early tumour resection have quicker recovery and shorter lengths of stay in the hospital. The inability to recognise a teratoma in analogous cases frequently leads to delayed neurological impairment, further supporting the use of screening pelvic imaging in female patients with autoimmune encephalitis [15].

Beyond individual treatment strategies, we warrant the necessity of a collaborative approach across specialities. Neurologists must recognise when neuropsychiatric symptoms warrant antibody testing and avoid premature psychiatric labelling, and psychiatrists must remain aware of autoimmune mimics to prevent inappropriate treatment with psychotropic medications alone [22]. Gynaecologists also have a critical role in identifying and managing teratoma-associated cases, ensuring that tumour removal occurs promptly. The intersection of these specialities determines the success of the intervention, and delayed recognition in any one area can impact patient outcomes. 

The full recovery of the patient in the current case, both neurologically and functionally, underscores the importance of early diagnosis and targeted treatment among patients with ovarian teratomas triggering anti-NMDA receptor encephalitis.

## Conclusions

This case reinforces several clinical lessons. First, in cases with young women presenting with unexplained neuropsychiatric symptoms, a multidisciplinary approach including neurologists, psychiatrists, and gynaecologists is required. Second, it emphasises the importance of including anti-NMDA receptor encephalitis early in the differential diagnosis, especially if initial investigations are inconclusive. Finally, the delayed improvement until after tumour resection demonstrates the value of early detection and surgical management of ovarian teratomas for optimal neurological recovery.
